# Issue Information

**DOI:** 10.1002/lrh2.10063

**Published:** 2019-10-21

## Abstract

No abstract is available for this article.

